# Case of Re-Union between Two Portions of the Finger, after They Had Been for a Considerable Time Separated

**Published:** 1821-02

**Authors:** Miles Marley

**Affiliations:** Member of the Royal College of Surgeons.


*Case of Re-union between two Portions of the Finger, after they had been for a considerable time separated.* By Mr. Miles Marley, Member of the Royal College of Surgeons.

A LAD, aged 11 years, whilst playing near some new buildings in the neighbourhood of Chelsea, had one half of the phalanx of the index-finger of the left hand severed off by a flag. He was brought to me about ten minutes after the accident; when I found the stump, as well as the whole of the hand, more especially the middle finger, very much bruised and lacerated. I immediately sent the friends of the boy to search for the separated part; and, during their absence, I was employed in clearing the hand from the dirt, &c. with which it was covered. The mother returned in about twenty minutes with the separated part, which was quite cold and of a livid colour. Having washed it, I brought the lacerated surfaces into contact by means of adhesive plaster, and desired that the arm might be kept completely at rest. It was examined on the fifth day, when perfect re-union had taken place. The nail came away in the course of eight days, and the inflammation that followed was so slight as not to require any attention. The second dressing was not applied till the tenth day, after which it was dressed every other day.

It is now nearly three months since the accident occurred: the lad has perfect motion and sense of touch in the finger, and the nail is almost wholly regenerated.

*Vigo-lane, Burlington-Gardens;*
*January 1st,* 1821.

